# Antihypertensive Effect of *Syzygium cumini* in Spontaneously Hypertensive Rats

**DOI:** 10.1155/2014/605452

**Published:** 2014-12-28

**Authors:** Rachel Melo Ribeiro, Vicente Férrer Pinheiro Neto, Kllysmann Santos Ribeiro, Denilson Amorim Vieira, Iracelle Carvalho Abreu, Selma do Nascimento Silva, Maria do Socorro de Sousa Cartágenes, Sônia Maria de Farias Freire, Antonio Carlos Romão Borges, Marilene Oliveira da Rocha Borges

**Affiliations:** ^1^Laboratory of Pharmacology, Department of Physiological Science, Federal University of Maranhão (UFMA), Avenida dos Portugueses 1966, Bacanga, 65080-805 São Luís, MA, Brazil; ^2^Department of Veterinary Medicine, State University of Maranhão (UEMA), 65055-970 São Luís, MA, Brazil

## Abstract

This study evaluated the *in vivo* potential antihypertensive effect of hydroalcoholic extract of *Syzygium cumini* leaves (HESC) in normotensive Wistar rats and in spontaneously hypertensive rats (SHR), as well as its *in vitro* effect on the vascular reactivity of resistance arteries. The hypotensive effect caused by intravenous infusion of HESC (0.01–4.0 mg/kg) in anesthetized Wistar rats was dose-dependent and was partially inhibited by pretreatment with atropine sulfate. SHR received HESC (0.5 g/kg/day), orally, for 8 weeks and mean arterial pressure, heart rate, and vascular reactivity were evaluated. Daily oral administration of HESC resulted in a time-dependent blood pressure reduction in SHR, with a maximum reduction of 62%. In the endothelium-deprived superior mesenteric arteries rings the treatment with HESC reduced by 40% the maximum effect (*E*_max⁡_) of contraction induced by NE. The contractile response to calcium and NE of endothelium-deprived mesenteric rings isolated from untreated SHR was reduced in a concentration-dependent manner by HESC (0.1, 0.25, and 0.5 mg/mL). This study demonstrated that *Syzygium cumini* reduces the blood pressure and heart rate of SHR and that this antihypertensive effect is probably due to the inhibition of arterial tone and extracellular calcium influx.

## 1. Introduction

More than one-fourth of the world's adult population suffers from arterial hypertension [1]. The objective of antihypertensive treatment is to achieve optimal blood pressure levels during therapy in order to reduce hypertension-related complications. Studies have shown that a reduction of 5 mmHg in systolic blood pressure (SBP) is associated with a 7% reduction in cardiovascular mortality [2, 3]. The research literature indicates that complementary and alternative medicine is an important potential field for the treatment of hypertension by contributing in reducing blood pressure levels and minimizing its complications [4].

*Syzygium cumini* L. Skeels (syn.* Eugenia jambolana* Lam.) is a tropical tree of the family Myrtaceae, which is popularly known as jambolan. The plant is extensively used for the treatment of different diseases, such as inflammation, constipation, obesity, urinary disorders, diabetes, and hypertension [5–9]. Pharmacological studies have suggested hypoglycemic and antihyperglycemic properties of this medicinal species [10–19]. Other experimental studies using different extracts of the plant suggest that* S. cumini* possesses antioxidant activity [20], antiatherosclerotic potential [21], and a cardioprotective effect [22–24].

Despite the biological potential of* S. cumini* as a pharmacological tool, studies validating its use as an antihypertensive agent are rare. In Brazil, it was observed that an aqueous extract of jambolan leaves reduced the blood pressure and presented diuretic effect in anesthetized normotensive rats [25, 26]. In addition, our group demonstrated an antagonistic action of hydroalcoholic* S. cumini* leaf extract on contractions induced by calcium in* in vitro* preparations of aorta from normotensive rats [27].

The use of medicinal plants as primary care is a common practice in Brazil. As a consequence, the Brazilian government has developed a program that compiled a list of medicinal plant species. On this list, called the National Register of Plants of Interest to the National Health System (Relação Nacional de Plantas de Interesse do Sistema Único de Saúde, RENISUS),* S. cumini* occupies the 64th position, but with the reservation that there are no studies or indication of use. In this respect, our research group has been working tirelessly to obtain data on this plant species and preclinical toxicological studies have shown that* S. cumini* exhibits no chronic toxicity [28], validating its safe use by the population.

The present study evaluated the antihypertensive effect of hydroalcoholic extract of* S. cumini* leaves in spontaneously hypertensive rats (SHR), as well as its hypotensive mechanism in* in vivo* and* in vitro* assays, in order to validate the use of this species as a pharmacological tool in ethnomedicine.

## 2. Materials and Methods

### 2.1. Plant

Leaves of* S. cumini* were collected from the campus of the Federal University of Maranhão (Universidade Federal do Maranhão, UFMA), São Luís, Brazil, in October 2012. A voucher specimen was identified and deposited in the herbarium of the “Professor Dr. Berta Lange de Morretes” Medicinal Plant Garden, UFMA (number 1069).

### 2.2. Preparation of the Hydroalcoholic Extract of* Syzygium cumini*

The leaves were dried at room temperature and pulverized. The crude extract was prepared by maceration of the leaf powder in 70% ethanol and concentration in a rotary evaporator under reduced pressure at a temperature below 60°C. The concentrated extract thus obtained was called the hydroalcoholic extract of* S. cumini* leaves (HESC) with a dry weight of 60 g, yield of 16.3% (Brazil, PI 1101652-3/18 of June, 2013) [29]. Phytochemical screening revealed the presence of hydrolyzable tannins, phenols, flavonoids, steroids, terpenoids, quinones, and saponins.

### 2.3. Animals

Male 12-week-old spontaneously hypertensive rats (SHR) and Wistar rats (*Rattus norvegicus*), weighing 250 to 300 g, obtained from the Animal House of UFMA were used. The animals were housed under controlled conditions of temperature (21 ± 2°C) under a 12 h light-dark cycle, with ration and water available* ad libitum*. After a period of adaptation, SHR were randomly divided into a control group and a group treated with HESC (0.5 g/kg/day, orally). Wistar rats were used as normotensive controls. The control groups received a similar volume of water (0.1 mL/100 g). The experimental protocols were approved by the Animal Research Ethics Committee of UEMA, Brazil (Protocol 17/2012).

### 2.4. Effect of HESC on the Blood Pressure of Normotensive Rats

Wistar rats were anesthetized with 20% urethane (0.8 g/kg, i.p.) and 1% pentobarbital sodium (40 mg/kg, i.p.). Next, tracheostomy was created and the right femoral vein was cannulated for administration of the drugs and extract. A bolus injection of heparin (30 IU) was given immediately after the venous access. The left carotid artery was cannulated for mean arterial pressure (MAP) recording. Pressure variations in the carotid artery were recorded with a pressure transducer (Mod F-60, Narco Biosystems, Inc., Houston, Texas, USA) connected to a Narcotrace 40 polygraph (Narco Biosystems, Inc., Texas, USA). After the surgical procedure, the hemodynamic variables were allowed to stabilize for 15 min before injection of the test substances. All drugs and the extract were dissolved in 0.9% NaCl (saline). Acetylcholine (ACh; 1.0 *μ*g/kg/i.v.) was chosen as the drug standard curve of physiological mechanisms.

The intravenous injections of HESC (0.01–4.0 mg/kg/i.v.) and acetylcholine did not exceed 0.1 mL over a period of 5 s and were followed by the application of 0.1 mL saline to wash the cannula. In addition, the participation of the cholinergic system was evaluated by injecting atropine (ATR; 1.0 mg/kg).

### 2.5. Effect of Prolonged Treatment with HESC on the Blood Pressure and Heart Rate of SHR

The MAP and heart rate of SHR were measured using the indirect tail-cuff plethysmography method [30]. According to this protocol, an appropriately sized occlusion cuff was placed around the tail of the animals and connected to a plethysmograph (LE 5001 Pressure Meter, Panlab, Cornella, Spain). A mean of three measurements was obtained for each animal. For blood pressure measurement, the animals were warmed up to 42°C for 5 min in a confinement cage. The animals were first submitted to a period of adaptation for 15 days before the experiments and only SHR with an SBP > 170 mmHg were selected for this study. The animals received a daily oral administration of water (0.5 mL/100 g) or HESC (0.5 g/kg) for 8 weeks. In addition, the weight of the animal and food intake were determined weekly and treatment of the animals was always preceded by blood pressure measurement.

### 2.6. Effect of Prolonged Treatment with HESC on Vascular Reactivity in SHR

Preparations of the mesenteric artery were obtained [30–32] and ring segments (3–5 mm) of the superior mesenteric artery were placed between stainless steel wires (50 *μ*m in diameter) and immersed in an organ bath chamber (5 mL) containing Krebs nutritive solution (118 mM NaCl, 5 mM KCl, 1.2 mM MgCl_2_, 1.2 mM NaH_2_PO_4_, 15.5 mM NaHCO_3_, 2 mM CaCl_2_, and 11 mM glucose, pH 7.4) at 37°C, equilibrated with 5% CO_2_/95% O_2_. The presence of endothelial function was confirmed by the capacity of acetylcholine (10 *μ*M) to induce more than 70% of relaxation of vessels precontracted with norepinephrine (NE, 10 *μ*M).

Changes in the isometric tension of the preparations were measured with an isometric force transducer (PowerLab, ADInstruments Pty. Ltd., Sydney, Australia). The preparations were first equilibrated under a tension of 1.0 g and washed at intervals of 10 min. After 60 min of successive washes, cumulative dose-response curves to NE (10^−9^ to 10^−4^ M) were obtained for SHR treated with HESC (0.5 g/kg/day).

### 2.7. *In Vitro* Effect of HESC on Contractions Induced by Norepinephrine or CaCl_2_ in the Mesenteric Artery of SHR

Spontaneously hypertensive rats were euthanized and superior mesenteric arterial rings were obtained [30–32] and rings without endothelium were used for this experiment. Thereafter, cumulative concentration-response curves to NE (10^−9^ to 10^−4^ M) were constructed in the absence or presence of HESC (0.1, 0.25, and 0.5 mg/mL).

To evaluate the antagonistic action of HESC against calcium, vascular tissue was stabilized with normal Krebs solution; after 30 min, the fluid of the preparation was replaced with Ca^2+^-free Krebs solution (60 mM K^+^, nominally Ca^2+^-free). After 30 min of successive washes, the basal tone was recovered, which permitted obtaining cumulative concentration-response curves to CaCl_2_ (10^−6^ to 10^−2^ M) in the absence or presence of HESC (0.1, 0.25, and 0.5 mg/mL).

The concentration necessary to elicit 50% of the maximum response (EC_50_) was determined using nonlinear regression analysis. The negative logarithms of the EC_50_ values (pD_2_) were used for statistical analysis. In the experiments involving high extracellular K^+^, Krebs solution containing 60 mM KCl was prepared by replacing an equimolar concentration of NaCl with KCl.

### 2.8. Statistical Analysis

The results were expressed as the mean ± standard error of the mean (SEM). One-way analysis of variance (ANOVA) followed by the Newman-Keuls posttest was used for multiple comparisons and Student's* t*-test for comparison of unpaired data. A *P* value < 0.05 was considered significant. Statistical analysis was performed using the GraphPad Prism 5.0 program.

## 3. Results

**Figure 1:**
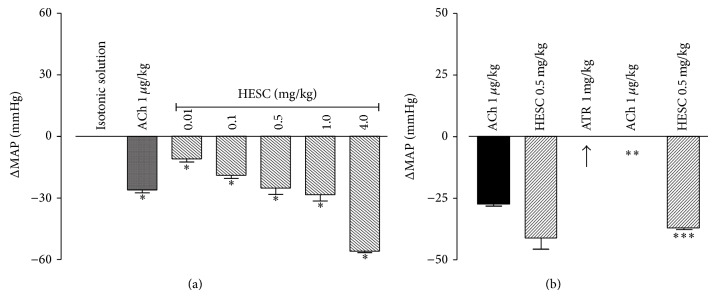
Effect of intravenous injections of the hydroalcoholic extract of* Syzygium cumini* leaves (HESC; 0.01 to 4.0 mg/kg) on the blood pressure of Wistar anesthetized rats. (a) Dose-dependent hypotensive effect of HESC. (b) Partial inhibition of the hypotension induced by HESC (0.5 mg/kg) by atropine (ATR). Acetylcholine (ACh, 1.0 μg/kg) was used as an agonist in the production of the hypotensive effect. The columns and vertical bars indicate the mean ± SEM, respectively (*n* = 5) *P* < 0.05  ∗ versus isotonic solution; ∗∗ versus ACh 1.0 µg/kg and ∗∗∗ versus HESC 0.5 mg/kg (ANOVA, Newman-Keuls test). MAP: mean arterial pressure.

The administration of HESC to anesthetized normotensive rats (baseline MAP: 98.6 ± 0.05 mmHg) caused a dose-dependent reduction in MAP of 11.2 to 58.3 mmHg. The hypotension produced was reversed rapidly, returning to baseline values within 1 to 10 min depending on the dose administered. The administration of isotonic solution (0.9% NaCl, 1 mL/kg) did not cause significant blood pressure changes (Figure 1(a)). The hypotensive effect of the average HESC dose (0.5 mg/kg) on the MAP of anesthetized rats was modified in part by pretreatment with atropine sulfate (1 mg/kg), a dose sufficient to promote total blockade of acetylcholine (1 *μ*g/kg) (Figure 1(b)).

**Figure 2:**
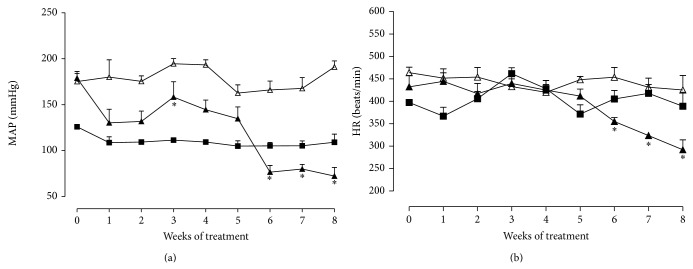
Effect of oral administration of the hydroalcoholic extract of* Syzygium cumini* leaves (HESC) for 8 weeks on the mean arterial pressure (MAP, a) and heart rate (HR, b) of spontaneously hypertensive rats. SHR control (Δ), SHR-HESC 0.5 g/kg (▲), and Wistar rats (■). Values are the mean ± SEM (*n* = 6-7). ^*^ *P* < 0.05 versus SHR control.

Figure 2 shows the effects of repeated oral administration of HESC (0.5 mg/kg/day) for 8 consecutive weeks on the MAP and heart rate of SHR. Baseline MAP and heart rate were, respectively, 126 ± 2.1 mmHg and 397.5 ± 6.4 bpm in Wistar rats (*n* = 7) and 175 ± 8.6 mmHg and 473.5 ± 8.4 bpm in SHR (*n* = 5–7). Oral HESC (0.5 mg/kg) significantly reduced the MAP and heart rate of SHR when compared to the group receiving only vehicle. This finding indicates a time-dependent antihypertensive effect, with this reduction ranging from 25% to 62% between weeks 4 and 8 of treatment (Figure 2(a)). In addition, a reduction in heart rate of 21% to 32% was observed between weeks 6 and 8 of treatment (Figure 2(b)).

**Figure 3:**
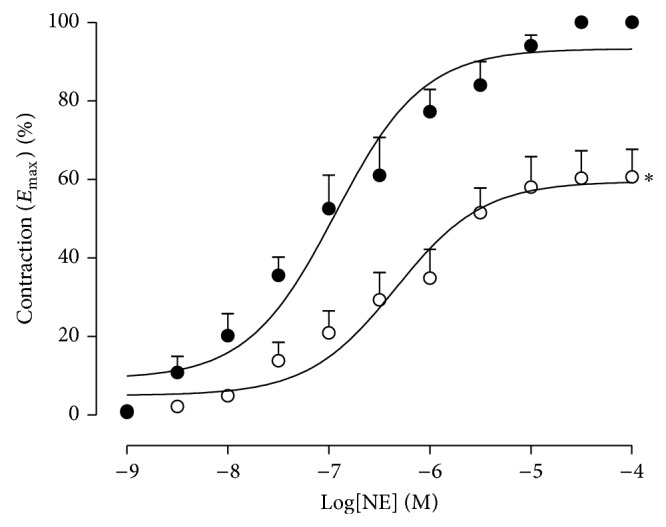
Cumulative dose-response curves to norepinephrine (NE) in isolated mesenteric arterial preparations from spontaneously hypertensive rats treated orally with the hydroalcoholic extract of* Syzygium cumini* leaves (HESC) (daily for 8 weeks). SHR control (●) and SHR-HESC 0.5 g/kg (о). Values are the mean ± SEM (*n* = 6-7). ^*^ *P* < 0.05 versus SHR control.

On the other hand, HESC altered the vascular reactivity of isolated mesenteric arteries from SHR treated for 8 weeks. The maximum effect (*E*_max⁡_) of the cumulative NE concentration-response curves was reduced by 40% (Figure 3) compared to the SHR control group, without the observation of changes in EC_50_ values (1.6 × 10^−7^ ± 3.0).

**Figure 4:**
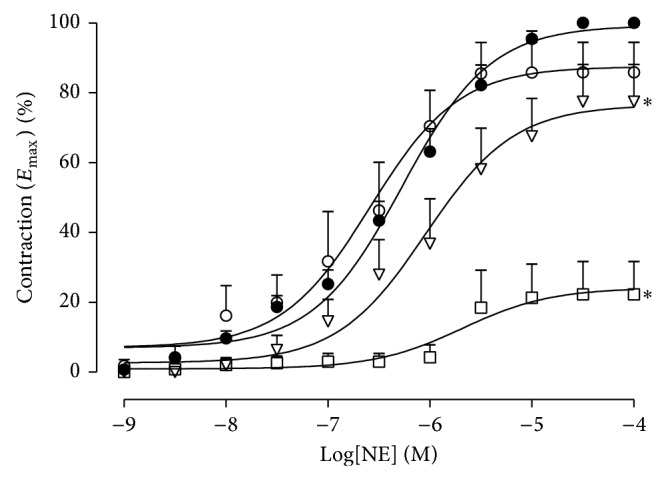
Cumulative dose-response curves to norepinephrine (NE) in isolated endothelium-deprived mesenteric arteries from spontaneously hypertensive rats. Control (●), HESC 0.1 mg/mL (o), HESC 0.25 mg/mL (∇), and HESC 0.5 mg/mL (□). Values are the mean ± SEM (*n* = 6-7). ^*^ *P* < 0.05 versus control.

*In vitro*, HESC at concentrations of 0.1, 0.25, and 0.5 mg/mL reduced the *E*_max⁡_ value for NE by 14%, 23%, and 78%, respectively. There was a displacement of the NE curves to the right, changing pD_2_ from 6.5 ± 0.1 to 5.68 ± 0.94, 6.17 ± 3.7, and 6.04 ± 0.2, respectively (Figure 4).

**Figure 5:**
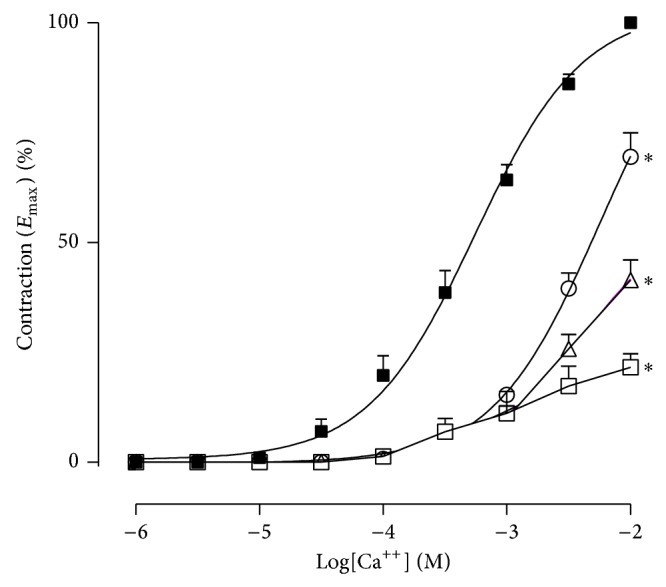
Cumulative dose-response curves to CaCl_2_ in isolated endothelium-deprived and depolarized mesenteric arteries from spontaneously hypertensive rats. Control (■), HESC 0.1 mg/mL (о), HESC 0.25 mg/mL (Δ), and HESC 0.5 mg/mL (□). Values are the mean ± SEM (*n* = 6-7). ^*^ *P* < 0.05 versus control.

In addition, contractions induced by CaCl_2_ in endothelium-deprived mesenteric arterial rings were reduced in a concentration-dependent manner after incubation with 0.1, 0.25, and 0.5 mg/mL HESC, with a reduction in maximum contraction to 75%, 53%, and 32% (*n* = 5–7), respectively (Figure 5). These effects were reversed after washing with Krebs solution.

## 4. Discussion

The present study showed an antihypertensive and bradycardic effect of* Syzygium cumini* in SHR. The hypotension promoted by HESC was modified by pretreatment with atropine sulfate, as observed in anesthetized rats, suggesting that muscarinic receptors play a role in the hypotensive mechanism of HESC. However, this effect may also be related in part to a reduction in peripheral vascular resistance as a result of the blockade of Ca^2+^ channels, as demonstrated in this study* in vitro*.

The finding that atropine sulfate partially modifies the hypotension produced by HESC (Figure 1(b)) indicates the participation of the cholinergic pathway, suggesting that the hypotensive effect of* Syzygium cumini* may be related to presence of cholinomimetic-muscarinic components [33, 34] (Figures 2(a) and 2(b)).

SHR were used in the present study which are a good experimental model to investigate factors involved in the pathogenesis of arterial hypertension, since these animals exhibit an increase in vascular reactivity accompanied by hyperresponsiveness to vasoconstrictor agonists [35]. The structural and functional vascular alterations that occur during hypertension are important pathological mechanisms that lead to the increase in blood pressure and are the targets of antihypertensive therapy [36, 37].

In this respect,* S. cumini* may be a potential candidate for antihypertensive therapy since HESC exerted a substantial blood pressure-lowering effect in SHR submitted to prolonged treatment (Figure 2(a)). The blood pressure reduction induced by the extract may be due in part to a decrease in peripheral vascular resistance, as demonstrated by the effect of HESC on NE-induced *E*_max⁡_ in superior mesenteric arterial rings of treated SHR. The participation of the sympathetic autonomic nervous system in the development and maintenance of different types of hypertension has been well established in the literature [38, 39].

Vasoconstrictor agents act on the regulation of intracellular calcium concentrations. Norepinephrine elicits a contractile response in vascular smooth muscle (VSM) mediated by the activation of G protein-coupled *α*-adrenergic receptors [40]. This activation, in turn, induces the formation of inositol triphosphate, corroborating the increase in intracellular calcium concentrations [41–44]. Calcium is therefore the primary regulator of tension in VSM [45, 46]. The maintenance of contraction depends on the influx of Ca^2+^ from the extracellular space through voltage- and/or receptor-operated calcium channels [47, 48]. Contractile agents such as KCl produce a significant increase in intracellular calcium through the activation of voltage-operated calcium channels. Therefore, contractions in VSM induced by depolarizing solutions (high K^+^ concentration and nominally Ca^2+^-free) have been frequently used to investigate the role of calcium in mechanisms that reduce vascular tone [49].

The present results showed that the* S. cumini* extract reduced the vasoconstrictor activity of calcium in a concentration-dependent manner in endothelium-deprived mesenteric arteries exposed to a depolarizing solution (60 mM KCl, nominally Ca^2+^-free). This finding indicates that HESC contains components that interfere with the responsiveness of VSM, probably acting on the regulation of intracellular calcium levels through voltage-operated calcium channels. Similar results of inhibition of contractile responses to calcium were found in previous studies with the hydroalcoholic extract of* S. cumini* by our laboratory in preparation of aortic rings isolated from normotensive rats in the presence of endothelium [27].

Many studies have shown that plant extracts containing flavonoids and/or triterpenes exert antihypertensive effects through the combination of the vasodilatory and antioxidant activities of these classes of compounds [37, 50]. The results of phytochemical screening showed that HESC is rich in these compounds, which may be responsible for the antihypertensive property of the plant.

In conclusion, the* in vivo* and* in vitro* results of the present study show that* Syzygium cumini* reduces the blood pressure and heart rate of SHR. This antihypertensive effect is probably due to inhibition of arterial tone by the blockade of extracellular Ca^2+^ influx. These effects can be attributed to the presence of flavonoids detected by phytochemical screening. The results may partially explain the traditional use of* S. cumini* for the treatment of disorders such as hypertension. However, further studies are needed to elucidate the possible antihypertensive mechanism of this medicinal plant.
